# Improving Mealtime Independence for People With Dementia Living in Nursing Homes: A Pilot Study

**DOI:** 10.1155/nrp/8948151

**Published:** 2025-10-08

**Authors:** Zhoumei Yan, Peta Drury, Ibrahim Alananzeh, Joel Zugai, Elizabeth Halcomb

**Affiliations:** ^1^School of Nursing, Faculty of Science, Medicine and Health, University of Wollongong, Wollongong, Australia; ^2^School of Healthcare, University of Wollongong, Dubai, UAE

**Keywords:** dementia, eating ability, eating difficulty, engagement and affect, Montessori-based activities, nursing home, nutrition, spaced retrieval

## Abstract

**Aim:**

This paper explores the impact of a Spaced Retrieval and Montessori-based activity (SPREMON) intervention on mealtime independence, mealtime engagement and affect (mood), and the nutritional status of people with dementia living in nursing homes.

**Methods:**

Quasi-experimental pilot study design. The SPREMON intervention consisted of 20 sessions delivered over 7 weeks. Data were collected at baseline, postintervention, and at 3-month follow-up, using the Edinburgh Feeding Evaluation in Dementia Scale, the Engagement of a Person with Dementia Scale, and the Mini Nutritional Assessment Short Form. Descriptive and inferential statistics were used to analyze the data.

**Results:**

Of the 20 consumer participants enrolled, 8 (40%) completed the intervention. The completed groups had significantly lower eating difficulty, significantly higher engagement and affect scores, and fewer comorbidities and polypharmacy than the dropouts at baseline. Engagement and affect and drink consumption changed significantly from pre- to postintervention. While positive trends were observed in eating difficulties and nutritional status scores within the completed group, these changes did not reach statistical significance.

**Conclusions:**

Delivering the SPREMON intervention via this pilot study demonstrates its feasibility and potential benefits, given the positive trends in eating independence, engagement, affect, and nutrition. Larger studies are needed to confirm these findings, assess who benefits most, and explore scalability.

**Implications:**

Combining SPREMON may improve eating independence, engagement and affect, and nutrition outcomes in people with dementia.

**Impact:**

SPREMON shows promise as a person-centered approach to enhance mealtime outcomes in dementia care, informing practice for health professionals, researchers, and care managers.

**Patient or Public Contribution:**

This intervention was co-designed with aged care professionals and consumer participants.

**Trial registration:**

Australian New Zealand Clinical Trial Registry: 12623001031651.

## 1. Introduction

Dementia is a neurodegenerative disease, leading to a decline in cognitive and physical functions, affecting an individual's ability to live independently [1]. It is a significant public health issue, impacting a growing number of people and placing a substantial strain on healthcare systems [2]. In 2021, 57 million people worldwide were living with dementia, and in 2019, 1.3 trillion dollars (USD) was spent on dementia care [2]. Additionally, there are 10 million new cases globally each year [2]. In Australia, around 15 out of every 1000 Australians are living with dementia [3]. Although most (65%) of these people are living in the community, some 35% require support in residential aged care settings [3]. Among those individuals living in residential aged care facilities (nursing homes), 84% have complex cognitive and behavioral needs, 76% require assistance in undertaking activities of daily living, and 61% have complex health care conditions [3].

Food and nutrition are fundamental human needs and are vital in maintaining the health and well-being of a person with dementia. However, an individual's ability to eat typically declines as dementia progresses, significantly impacting their food and nutrient intake [4, 5]. Poor nutritional status is also associated with faster dementia progression, greater functional decline, and higher mortality rates [6]. People with dementia who are malnourished are three to four times more likely to experience severe dementia and death than those with adequate nutritional status [6]. A recent review found that the prevalence of malnutrition among people with dementia living in nursing homes was 27%, and nearly 60% of this cohort was at risk of malnutrition [7]. This underscores the pressing need to optimize self-eating abilities and increase food and nutrient intake among people with dementia living in nursing homes.

## 2. Background

Spaced Retrieval and Montessori-based activities (SPREMON) have gained considerable attention over the past decade for addressing eating difficulties and enhancing the nutritional status of people with dementia [8–10]. The Montessori method, originally developed for early childhood education, is based on key principles such as a person-centered learning approach, respect for individual dignity, equality, autonomy, and consideration of personal preferences, strengths, and limitations [11]. This approach is increasingly considered valuable in aged and dementia care [10]. By emphasizing respect, dignity, and equality, it fosters environments where people with dementia can engage in meaningful activities, promoting independence and a sense of purpose [11].

Spaced Retrieval, on the other hand, is a cognitive learning technique that involves recalling information at progressively increasing intervals [12]. This approach is grounded in the idea that information is more effectively retained when reviewed or recalled multiple times, with each review in increasing time intervals [12]. This method has previously been used to enhance the learning and information retention abilities of people with dementia [13].

A recent review highlighted the synergistic effect of combining Spaced Retrieval, to train procedural memory, with Montessori-based activities that enhance motor skills related to eating in people with dementia [10]. This combination has been shown to be more impactful than either approach alone in improving self-feeding ability and nutritional outcomes [8, 9, 14, 15]. Collectively, these studies suggest that integrating procedural memory with motor-based activity training not only reinforces cognitive learning but also promotes functional independence (eating) in this population [8, 9, 14, 15].

However, these previous studies have been conducted in Asian cultures and focused primarily on supporting people with dementia to feed themselves using a spoon. Western eating customs, eating with a knife, fork, and spoon, remain largely unexplored [10]. Therefore, this pilot study sought to evaluate the impact of the the SPREMON intervention intervention on mealtime independence, mealtime engagement and affect, and the nutritional status of people with dementia living in Australian nursing homes using Western-style eating etiquette.

## 3. Methods

### 3.1. Design

This quasi-experimental pilot study involved a single group of residents with multiple time-point assessments to measure changes in participants' eating difficulties, engagement and affect, and nutritional status. As it was not feasible to randomize participants, a quasi-experimental approach was chosen to enable the measurement of the effects of the intervention on key outcomes. The detailed study protocol has been published elsewhere [16]. The implementation of the intervention was supported by a multicomponent strategy to promote acceptability, feasibility, and potential scalability of the intervention. This included a co-design approach with aged care professionals to ensure contextual fit; strong organizational support through endorsement from management and integration into the facilities' activity schedules; appointment of staff champions to assist with recruitment and coordination; and a flexible delivery model accommodating individual preferences and pandemic-related restrictions . This paper was informed by the TREND statement [17].

### 3.2. Setting and Sample

Three nursing homes in regional New South Wales, Australia, owned by the same organization, were recruited for this study. Each facility accommodates 120–140 residents and employs approximately 100–120 staff, including service managers, Baccalaureate-prepared (or equivalent) Registered Nurses, Diploma-prepared Enrolled Nurses, Certificate-qualified nursing assistants, allied health professionals, and catering staff. Catering staff are responsible for meal preparation and delivery of meals to residents, either in communal dining areas or individual rooms, depending on residents' preferences. Their role may also include setting up meals by opening lids, arranging cutlery, and placing items within reach. Direct mealtime assistance, such as cutting food or spoon-feeding, is primarily provided by Enrolled Nurses and Nursing Assistants, under the supervision of Registered Nurses. Staff members are allocated each shift to support residents who require feeding assistance. In contrast, residents able to eat independently typically sit together at larger communal tables. As this study was conducted during the COVID-19 pandemic, changes were occasionally implemented within participating facilities regarding visitor attendance and residents' common dining areas. During acute outbreaks of COVID-19, visitors were not permitted, and meals were served in residents' rooms, with staff providing mealtime assistance according to the residents' needs.

Nursing home managers delegated a staff member to assist with participant recruitment. This study champion screened electronic health records and created a list of potential participants. Next, the PhD candidate (ZY) briefly observed the eating behaviors of the potential participants to confirm if they met the inclusion criteria.

### 3.3. Participants

Residents were eligible to participate if they: (a) had a formal diagnosis of dementia or cognitive impairment documented in their medical records; (b) required ongoing verbal prompts to continue eating; (c) were deemed safe for oral intake by a registered nurse or speech pathologist; (d) were physically able to use standard Western utensils (knife, fork, and spoon) for eating; and (e) were able to remain seated in a chair for approximately 45 min.

Residents were excluded if they (a) required complete staff assistance with feeding during meals; (b) could not speak or comprehend English; or (c) presented with severe behavioral disturbances or long-standing psychiatric conditions unrelated to dementia, such as schizophrenia, personality disorders, or psychotic illnesses.

If residents met the inclusion criteria, the study champion contacted their guardians to seek permission for their participation in the study. Given the impact of the COVID-19 pandemic on nursing home visitors during the recruitment period, the study champion telephoned each guardian on up to two occasions to seek their consent. If no response was achieved from the calls, the study champion then sent an email providing information about the study, followed by one reminder email.

### 3.4. Intervention

The SPREMON intervention was developed based on previous literature [8, 9, 14, 15] and using a co-design process with Australian aged care professionals [18]. The intervention combined Spaced Retrieval to train procedural memory and Montessori-based activities to train the motor skills of eating [16]. The intervention was delivered by the PhD candidate (ZY) in the activity rooms of three participating nursing homes over 20 sessions, each lasting 45 min and held on Monday, Wednesday, and Friday mornings. This schedule aligned with the facilities' regular recreational programing. The number and duration of sessions were kept consistent across all three sites. However, participants were free to leave any session at any time should they choose to do so. While the activity was initially aimed to be conducted in small groups of three to five people; individual activities were also conducted due to personal preferences and the impact of COVID-19.

### 3.5. Data Collection

Demographic data were collected from the medical records. These data included gender, age, type of dementia, and time since diagnosis. Additionally, a clinical assessment of dementia severity (Mini-Mental State Examination) [19], comorbidity and polypharmacy score [20], and activities of daily living (Barthel Index) [21] were undertaken before study enrollment using usual clinical processes.

Outcomes data were collected at baseline, postintervention, and at 3 months following the intervention by the PhD candidate (ZY) by observing participants' mealtime behaviors at a single meal. These timepoints were chosen to immediately follow the intervention and a medium term timepoint following intervention conclusion. The primary outcome, eating difficulties, was measured by the Edinburgh Feeding Evaluation in Dementia Questionnaire (EDFED-Q) [22]. EDFED-Q scores range from 0 to 20, with higher scores indicating greater eating difficulties [22]. Secondary outcomes were measured using the Engagement of a Person with Dementia Scale (EPWDS) [23] and the Mini Nutrition Assessment-Short Form (MNA-SF) [24] to measure engagement and affect, and nutrition, respectively. The EPWDS scores range from 10 to 50, with higher scores reflecting greater engagement and more positive mood states [23]. Scores on the MNA-SF indicate varying levels of nutritional status, with scores of 0–7 indicating malnutrition, 8–11 indicating risk of malnutrition, and 12–14 reflecting normal nutritional status [24]. The percentage of food consumption was measured by visual estimation.

### 3.6. Data Analysis

Data were initially recorded on hard copy data forms, and then entered into a Microsoft Excel spreadsheet by the PhD candidate (ZY). They were subsequently imported into IBM SPSS Statistics Version 28 for analysis. Participants who completed > 50% of the intervention sessions or who had all outcome data available were considered to have completed the intervention. The demographic information and baseline data were compared to explore similarities and differences between those who did not complete the intervention (dropouts) and the completed group.

Descriptive statistics, including means and standard deviations (SD), were used to present continuous data (e.g., age, years since diagnosis). The mean and standard error (SE) were used to present key study outcomes, including eating difficulties scores, engagement and affect, and nutrition scores. SE is commonly used in studies with small sample sizes because it provides an estimate of the variability of the sample mean and indicates the precision of the estimate [25]. A paired *t*-test was used to compare mean differences between the completed and dropout groups. The Friedman test was used to assess changes in key outcomes over time. A value of *p* < 0.05 was considered statistically significant.

### 3.7. Ethical Considerations

Ethical approval was obtained from the University of Wollongong Human Research Ethics Committee (Approval number: 2023/268). As participants had dementia and variable cognitive capacity, the project champion informed family members about the study and obtained their consent for participation. Verbal consent was also obtained from individual participants before each session.

## 4. Results

### 4.1. Participant Recruitment and Retention

**Figure 1:**
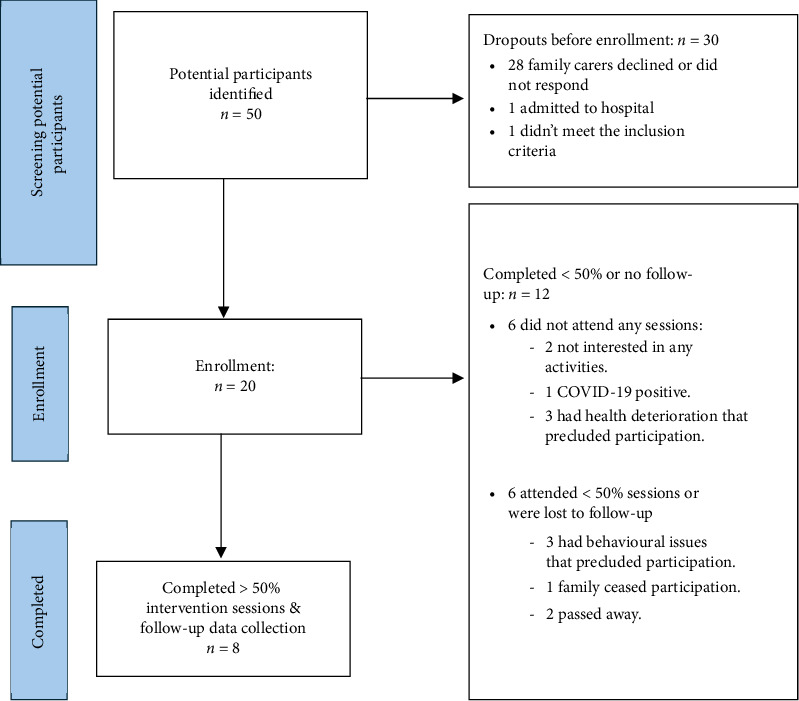
Recruitment flow diagram.

Of the 50 potential participants identified, more than half of the family members (*n* = 28, 56.0%) were either unable to be contacted or declined to provide consent for study participation (Figure 1). Additionally, one resident (2.0%) was admitted to the hospital, and another (2.0%) was excluded due to dysphagia. Therefore, 20 individuals (40.0%) were enrolled in the study.

Of those enrolled, 6 (30.0%) participants did not attend any sessions, and 6 (30.0%) participants did not complete > 50% of the intervention sessions or passed away (dropout group). Participants did not attend sessions for a range of reasons, including refusal to participate, admission to hospital, and health deterioration (including infectious conditions necessitating isolation). This left eight participants (40.0%) who completed the intervention and were available for follow-up (completed group). No adverse events occurred during the intervention and follow-up period.

### 4.2. Completed Participants

All participants who completed the study were female, with a mean age of 88.3 ± 8.5 years (Table 1). Most (*n* = 6; 75%) had been diagnosed with dementia within the past 1 to 3 years.

There was no significant difference in age between those who completed the intervention and the dropout group (Table 2). However, the dropout group had been diagnosed with dementia for a significantly longer period (*p*=0.008) and a significantly higher comorbidity and polypharmacy score (*p*=0.033) than the completed group. The completed group also had significantly lower feeding difficulties scores (*p*=0.022) and significantly higher engagement and affect scores (*p*=0.013) than the dropout group. No significant differences were found between the completed group and the dropout group for nutrition score, food consumption, eating time, or body weight.

### 4.3. Activity Participation and Performance

Participants in the completed group attended a mean of 16.6 sessions (83.75% attendance), participated in Montessori-based activities for an average of 724.4 out of 900 minutes (81% participation rate), and achieved an average Spaced Retrieval accuracy rate of 93% (Table 3). The dropout group attended between 1 and 12 sessions (mean: 4.8 sessions) and participated in Montessori-based activities for 8–375 minutes (mean: 165.5 minutes), achieving an average Spaced Retrieval response accuracy rate of 75.5%.

### 4.4. Completed Group Outcomes

Significant improvements were observed for the completed group in both mean EPWDS scores and drink consumption (Table 4). The mean EPWDS score increased from 37.0 preintervention to 46.8 postintervention and remained stable at 46.3 at the 3-month follow-up (*p*=0.018). Drink consumption showed no change pre- and postintervention, remaining at 72%, but significantly increased to 100% at the 3-month follow-up (*p*=0.034). Although the trends in mean EDFED-Q scores, mean body weight, mean eating time, and mean MNA-SF scores were favourable, these did not reach statistical significance. Similarly, the mean percentage of main dish and dessert consumption was higher than baseline at the end of the intervention and the 3-month follow-up, but these differences were also not statistically significant.

### 4.5. Individual Outcomes

**Figure 2:**
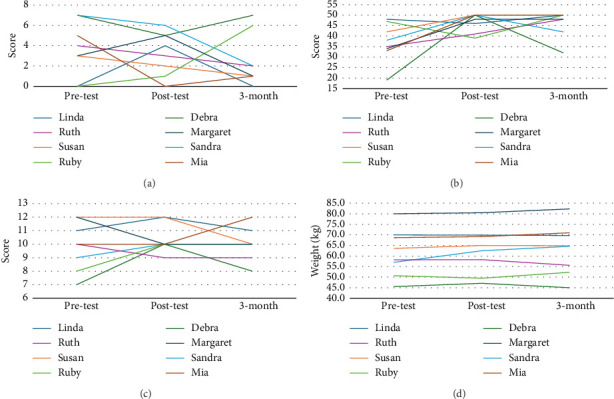
Individual outcome trends. (a) Eating difficulties score. (b) Engagement and affect scores. (c) Nutrition scores. (d) Body weight.

Given the small sample size, individual outcome trends were also examined. Baseline EDFED-Q scores varied among participants (Figure 2(a)). After the intervention, five (62.5%) participants had lower EDFED-Q scores, whereas three (37.5%) participants had higher scores compared to baseline. At the 3-month follow-up, five (62.5%) participants had lower EDFED-Q scores, two (25%) participants had no change, and one (12.5%) participant had a higher score at baseline.

Following the intervention, there was considerable improvement in EPWDS scores (Figure 2(b)). All but two participants (75%) showed an increase in EPWDS scores after the intervention. However, by the 3-month follow-up, all participants had a higher EPWDS score than at baseline. There was less variation in MNA-SF scores, with only a shift of between 0 and 3 points (mean 1.25 points postintervention and 1.38 points at the 3-month follow-up) per participant from baseline (Figure 2(c)). Postintervention, four participants (50%) increased their nutrition score, and two (25%) had no change. Three of the four participants sustained this increased nutrition score at the 3-month follow-up. In terms of body weight, five (62.5%) of the eight participants had an increased weight at the 3-month follow-up, gaining between 1.3 and 7.6 kg (Figure 2(d)). In contrast, three (37.5%) participants experienced weight loss, ranging from 0.3 to 2.6 kg.

## 5. Discussion

This pilot study evaluated the impact of a co-designed Spaced Retrieval and Montessori-based intervention that incorporated Western-style eating etiquette. Organizational support including committed management, provision of resources, and staff engagement, was essential to the project's implementation. Staff played a critical role by prioritizing assistance with residents' morning routines and escorting them to the SPREMON activities. In this study, the intervention was delivered by the PhD candidate (ZY); however, future large-scale implementation could be led by facility staff. This approach aligns with existing literature highlighting the importance of staff engagement in eating interventions [26]. The COVID-19 pandemic also posed challenges for intervention delivery. Social distancing restrictions limited opportunities for small-group activities and communal dining. Given that environmental factors have been shown to influence eating dependence in people with dementia [27], the extent to which these restrictions affected the intervention and its outcomes remains uncertain.

Study findings reveal positive trends in reducing eating difficulties, improving engagement and affect, and enhancing nutritional status among people with dementia following the SPREMON intervention. This demonstrates the potential of this approach to enhance mealtime independence, experience and nutrition for this cohort. However, the study also demonstrated key challenges in recruiting and retaining participants in the trial. This suggests a need for further investigation of the intervention and its optimal translation, both for broader testing of its effectiveness and within standard clinical care.

A significant improvement in engagement and affect scores was observed among the participants who completed the study. This finding aligns with international studies, which reported increased activity engagement and improved mood among people with dementia following participation in Montessori-based activities [28–30]. Interestingly, increased social and activity engagement in nursing homes was also associated with a reduction in both the number of individuals using psychotropic medications and the total number of such medications prescribed [30]. These findings suggest that Montessori-based activities can effectively address the social engagement needs of people with dementia living in nursing homes, and also contribute to reduced reliance on psychotropic medications among this population.

While the trend in other outcomes, such as mean EDFED-Q scores, mean body weight, mean eating time, and mean MNA-SF scores, was favorable, it did not reach statistical significance. Notwithstanding the small sample size, achieving a significant change in this group is challenging. Dementia involves progressive cognitive and physical decline, posing ongoing challenges to maintaining independence among this cohort [31]. This underscores the concept that clinically meaningful changes may be more relevant than statistical significance when evaluating intervention outcomes in this population [32, 33]. Since dementia involves an irreversible decline in cognitive and physical function, the maintenance of cognitive and physical abilities or slight improvements can be considered positive outcomes for this kind of intervention [1, 31]. Therefore, even in the absence of statistically significant results, the observed favorable trends highlight the potential of the SPREMON intervention to support functional maintenance and improve quality of life in people with dementia. Further investigation in larger trials across various facilities is needed to explore which people might benefit most from this kind of intervention and evaluate its broader impact on clinical outcomes. A key component of such further trials is the timing of outcome measures and the duration of the intervention and follow-up. Palese et al. [34] has demonstrated that the effects of eating interventions in dementia decrease when the intervention is ceased and require continuity to maintain gains. Therefore, there is a need to consider both how outcomes are measured and whether intervention activities are embedded within routine care to promote their sustainability.

Recruiting people with dementia into research poses significant challenges, often resulting in low enrolment and high dropout rates [35]. In this study, 60% of potential participants did not enrol, largely due to difficulties contacting family members and obtaining their consent. This was complicated by the impacts of COVID-19 on both the wider community and nursing homes in particular [36]. Family carers play a vital role in decision-making for people with dementia as their legal guardians [37]. Many studies have identified family consent as a critical factor influencing participation, especially when individuals with dementia have diminished capacity to provide informed consent [38–40]. Some family members may be hesitant to consent to participation due to concerns about physical or emotional burden, a lack of perceived benefit, or ethical concerns [38, 41]. Others may be difficult to contact, particularly when the person with dementia is living in a nursing home and their family members live far away or are occupied with other family and work responsibilities [42]. These barriers highlight the importance of building trust with families, clearly communicating the research aims and potential benefits, and implementing flexible, ethically sound consent procedures to improve recruitment and participation in dementia research.

Beyond the challenges in recruitment, this study found that it was difficult to retain people with dementia in the intervention. This has implications for future trials that may seek to recruit large sample sizes or randomize participants. Comparing those who were retained and those who dropped out provided some insight into the possible reasons for this. The dropout group had significantly higher eating difficulties, comorbidity, and polypharmacy scores, a significantly lower engagement and affect scores at baseline, as well as a significantly longer duration since dementia diagnosis. This highlights the need to target interventions carefully to individual characteristics. Additionally, it suggests that seeking to recruit residents earlier in the dementia trajectory may optimize the impact of the intervention. These findings are consistent with previous studies showing that eating performance is associated with cognitive and physical function, the use of psychotropic medications and comorbidities [5, 43]. These findings suggest that participants with better cognitive and physical function may be more likely to engage with and benefit from interventions [5, 43]. Therefore, future implementation efforts should consider screening for factors such as cognitive impairment level, comorbidity, and polypharmacy to identify individuals most likely to participate in and benefit from such programs. While the intervention was devised using co-design with aged care experts [18], the duration and number of sessions within the intervention may have contributed to difficulties in retention. Considering how the program is delivered and exploring the number and frequency of sessions required to achieve an effect is essential in future work. Additionally, adapting intervention strategies to accommodate individuals' preferences and needs, and promoting broader family involvement may improve retention and effectiveness [10].

### 5.1. Limitations

This study adopted a person-centered approach by tailoring activities to each participant's cognitive and physical abilities, as well as their personal preferences. Additionally, as an adaptation of work from Taiwan, the intervention was grounded in evidence-based practice. The SPREMON intervention was modified using a co-design approach to suit Western-style dining etiquette, particularly the use of a knife, fork, and spoon [18]. This culturally relevant adaptation supports both the practicality and replicability of the intervention.

Despite its strengths, the study has several limitations. It was conducted within a single organization, which may limit the generalizability of findings to other organizations with different care practices or resources. While this study adopted a quasi-experimental approach, other study designs could have been used to generate a higer level of evidence. Recruitment and retention challenges also impacted the study due to unavoidable factors such as proxy consent and health deterioration among the people with dementia. The modest sample size may have affected the statistical power of the findings. Therefore, individual trends were shown to provide a deeper picture of the outcomes. Given resource constraints, outcomes were measured by a researcher at the participating facility during a single lunchtime, which may not fully reflect overall eating behaviors or mealtime experiences. Additionally, the researcher undertaking the assessments also led the conduct of the study, which may have introduced potential bias. While a 3-month follow-up was selected as a medium-term outcome point in this study, the use of other time points may have yielded different findings.

### 5.2. Implications

The study findings indicate that the SPREMON intervention has the potential to improve mealtime independence, mealtime experience, and nutritional status of people with dementia living in nursing homes. However, the current program was challenging to implement due to the need to seek family consent and the complex health conditions of this cohort. Future work should use larger sample sizes across various institutions to consider the impact of the intervention on clinical outcomes. Additionally, the structure and delivery of the program need to be investigated to ascertain the frequency and number of sessions required to achieve an impact on outcomes and understand what is feasible within this cohort. Finally, future work needs to consider and identify which people living with dementia would benefit most from this type of intervention.

## 6. Conclusion

This pilot study demonstrated the potential benefits of the SPREMON intervention in enhancing mealtime independence, engagement and affect, and nutritional status for people with dementia living in nursing homes. Despite a modest sample size and challenges with recruitment and retention, the findings suggest that participants who completed the intervention experienced significant improvements in engagement and affect, along with positive trends in reducing eating difficulties and improving nutrition, though these did not reach statistical significance. A key strength of the intervention was its person-centered and culturally adapted design, which contributed to its relevance and acceptability in practice. Differences in dementia duration, comorbidity, and polypharmacy between the completed and dropout groups also highlight the importance of considering individual health factors when implementing such interventions. These findings underscore the need for carer-inclusive recruitment strategies and organizational support to improve participation. Future large-scale studies are needed to further explore the activity design, mechanisms of change, and scalability of SPREMON across diverse aged care settings.

## Figures and Tables

**Table 1:** Participant demographics.

Variables	Dropout group (*n* = 12) mean ± SD		Completed group (*n* = 8) mean ± SD		*t*	df	*p*
	*n*	%	*n*	%			
Gender							
Female	10	83.3	8	100			
Male	2	16.7	0	0			
Age (years)	85.6 ± 6.2		88.3 ± 8.5		0.818	18	0.424
75–85	6	50.0	2	25.0			
86–96	6	50.0	5	62.5			
≥ 97	0	0	1	12.5			
Type of dementia							
Alzheimer's	6	50.0	3	37.5			
Vascular	0	0	1	12.5			
Non specified	4	33.3	4	50.0			
Mixed dementia	2	16.7	0	0			
Years since diagnosed	4.4 ± 2.0		2.3 ± 1.1		−0.960	18	0.008^∗^
1–3	5	41.7	6	75.0			
4–6	5	41.7	2	25.0			
7–9	2	16.7	0	0			
Comorbidity and polypharmacy score	35.3 ± 8.0		26.0 ± 10.0		−2.311	18	0.033^∗^
0–7 (mild)	0	0	0	0			
8–14 (moderate)	0	0	1	12.5			
15–21 (severe)	1	8.3	2	25.0			
> 22 (morbid)	11	91.7	5	62.5			
ADL score	20.0 ± 10.0		21.3 ± 10.6		0.261	18	0.797
0–20	8	66.7	4	50.0			
21–40	4	33.7	4	50.0			
MMSE score	5.3 ± 6.4		12.1 ± 9.5		1.902	18	0.073
0–10 (severe)	9	75	5	62.5			
11–20 (moderate)	3	25	0	0			
21–26 (mild)	0	0	3	37.5			
Mobility							
Walker	7	58.3	4	50			
Wheelchair	5	41.7	4	50			

*Note:* ADL score, activity of daily living score; MMSE score, Mini-Mental State Examination.

^∗^ *p* < 0.05. Permission has been obtained from PAR to use the MMSE form.

**Table 2:** Baseline measures.

Variables	Dropout group (*n* = 12)	Completed group (*n* = 8)	*t*	df	*p*
	Mean ± SE	Mean ± SE			
EDFED-Q	8.0 ± 1.3	3.6 ± 1.0	−2.503	18	0.022^∗^
EPWDS	26.9 ± 2.1	37.0 ± 3.3	2.748	18	0.013^∗^
MNA-SF	8.8 ± 0.9	9.9 ± 0.6	0.927	18	0.366
Food consumption (%)					
Main	54.2 ± 8.6	62.5 ± 13.4	0.551	18	0.589
Dessert	79.2 ± 8.0	87.5 ± 12.5	0.589	18	0.563
Drink	79.2 ± 8.6	71.9 ± 8.8	−0.571	18	0.575
Eating time (minutes)	28.7 ± 2.8	30.0 ± 2.0	0.354	18	0.728
Body weight (kg)	62.2 ± 5.2	61.7 ± 4.0	−0.061	18	0.476

*Note:* EDFED-Q, eating difficulties score; EPWDS, engagement and affect score, MNA-SF, nutrition score.

^∗^ *p* < 0.05.

**Table 3:** Activity participation and performance.

	Participant	Sessions attended (total 20)	% attendance	Engagement in Montessori-based activity (45 min/session, total 900 min)	Montessori-based activity participation (%)	Total spaced retrieval response accuracy (%)
Completed group	Linda	20	100	900	100	91
	Susan	20	100	880	98	100
	Debra	19	95	855	95	100
	Sandra	13	65	585	65	100
	Ruth	20	100	865	96	81
	Ruby	17	85	765	85	84
	Margaret	14	70	530	59	91
	Mia	11	55	415	46	98

	Mean ± SD	16.6 ± 3.6	83.7 ± 18.1	724.4 ± 187.6	80.5 ± 20.1	93.1 ± 7.6

Dropout group	D 1	12	60^#^	375	42	75
	D 2	7	35^#^	275	31	100
	D 3	4	20	180	20	100
	D4	3	15	90	10	93
	D5	1	5	8	1	10
	D6	2	10	65	7	75

	Mean ± SD	4.8 ± 4.1	24.2 ± 20.4	165.5 ± 139.1	18.5 ± 15.63	75.5 ± 34.1

^#^Participants passed away during the intervention period.

**Table 4:** Outcomes.

	Mean ± SE			*F* ^a^	*p*	Effect size^b^
	Baseline	Post-test	3 months			
EDFED-Q	3.6 ± 1.0	3.3 ± 0.8	3.0 ± 0.9	0.800	0.670	0.050
EPWDS	37.0 ± 3.3	46.8 ± 1.6	46.3 ± 2.3	8.069	0.018^∗^	0.504
MNA-SF	9.9 ± 0.6	10.4 ± 0.4	10.0 ± 0.4	1.040	0.595	0.065
Food consumption (%)						
Main	62.5 ± 13.4	81.3 ± 7.8	68.8 ± 10.3	1.652	0.438	0.103
Dessert	87.5 ± 12.5	100.0 ± 0	90.6 ± 9.4	2.000	0.368	0.125
Drink	71.9 ± 8.8	71.9 ± 12.0	100.0 ± 0	6.778	0.034^∗^	0.424
Eating time (mins)	30.0 ± 2.0	31.8 ± 3.3	32.5 ± 5.1	0.643	0.725	0.040
Body weight (kg)	61.7 ± 4.0	62.8 ± 3.9	63.2 ± 4.2	1.750	0.417	0.109

*Note:* EDFED-Q, eating difficulties score; EPWDS, engagement and affect score, MNA-SF, nutrition score.

^a^Friedman's ANOVA.

^b^Kendall's W.

^∗^ *p* < 0.05.

## Data Availability

The data that support the findings of this study are available on request from the corresponding author. The data are not publicly available due to privacy or ethical restrictions.
